# Experimental investigation of tensile properties and fracture behavior of ophthalmic sutures under straight, knotted, and looped configurations: Vicryl, nylon, and polypropylene

**DOI:** 10.1371/journal.pone.0343682

**Published:** 2026-02-27

**Authors:** Rasmiranjan Dalai, Vinay S, Sai N.S.H.C, Vineet J, Ramji M, Sayan B, Viswanath C

**Affiliations:** 1 Department of Mechanical and Aerospace Engineering, Indian Institute of Technology, Hyderabad, India; 2 Brien Holden Eye Research Centre, Hyderabad Eye Research Foundation, LV Prasad Eye Institute, Hyderabad, India; University of Padova: Universita degli Studi di Padova, ITALY

## Abstract

The deformation and failure behavior of sutures play a vital role in the surgical outcomes of soft tissue encountered in ophthalmic surgeries. In the present study, the mechanical characteristics of ophthalmic sutures, including 6–0/7–0/8–0 braided-Vicryl, 8–0/9–0/10–0 monofilament-Nylon, and 9–0/10–0 monofilament-Polypropylene sutures, are investigated under straight configuration (SC), knotted configuration (KC), and looped configuration (LC). Uniaxial tensile tests of sutures were conducted according to ASTM D2256/D2256M-21 at a strain rate of 30 mm/min with a gauge length of 120 mm. The initial stiffness, yield strength, breaking strength, elongation at break, resilience, and work of rupture were obtained from the experiments. Vicryl and Nylon sutures exhibited an *S*-shaped load vs. extension response, while Polypropylene exhibited an *R*-shaped profile. The sutures in SC displayed a smooth load vs. extension curve. Whereas the sutures in KC and LC showed a small load drop in the inelastic region during the full development of the knot. Further, fractography of failed surfaces was investigated using SEM to characterize the fracture behavior. Vicryl sutures predominantly failed in the high-strain-rate tensile break, whereas Nylon and Polypropylene sutures failed in the low-strain-rate tensile break in the SC. Meanwhile, the sutures in KC and LC experienced mainly tensile-shear failure due to the presence of knots. The present study’s outcomes help surgeons select an appropriate suture for a given ophthalmic tissue, thereby achieving a better surgical outcome based on its mechanical properties.

## 1. Introduction

The mechanical behavior of sutures plays a pivotal role in the wound healing process of the sutured tissue or incisions. Sutures aid in faster healing, resulting in reduced scarring and improved functionality [1]. They minimize the risks of wound breakdown and infections from pathogenic microorganisms while providing sufficient mechanical support. The healing of the sutured vascular tissue (Skin, Sclera, Aorta, etc) is quite different from that of sutured avascular tissues (Cornea, Ligaments, etc). Sutures in vascular tissues prevent them from breaching the epithelium, bearing the connecting tissue, and reducing blood loss by stabilizing the clots [1]. In corneal tissues, sutures facilitate rapid wound repair by inducing a high inflammatory response that stimulates keratocyte activation, ensures good apposition, and promotes the formation of fibroblast orientation perpendicular to the wound [2,3]. The mechanical properties of the sutures are crucial in selecting the proper suture sizes for a given tissue type. The various grades of sutures commonly used for soft tissues are listed in Table 1. The lower the suture size, the higher the tension the suture can handle.

**Table 1:** Commonly Used Sutures for Soft Tissues [4–10].

Size(USP)	Ophthalmic	Non-Ophthalmic
**6−0**	Eyelid skin,	Skin
**7−0**	Extraocular muscles	Facial muscles
**8−0**	Sclera and Conjunctiva	Anastomosis
**9−0**	Limbus and Iris	Microvascular tissue
**10−0**	Cornea	Nerve

**Fig 1:**
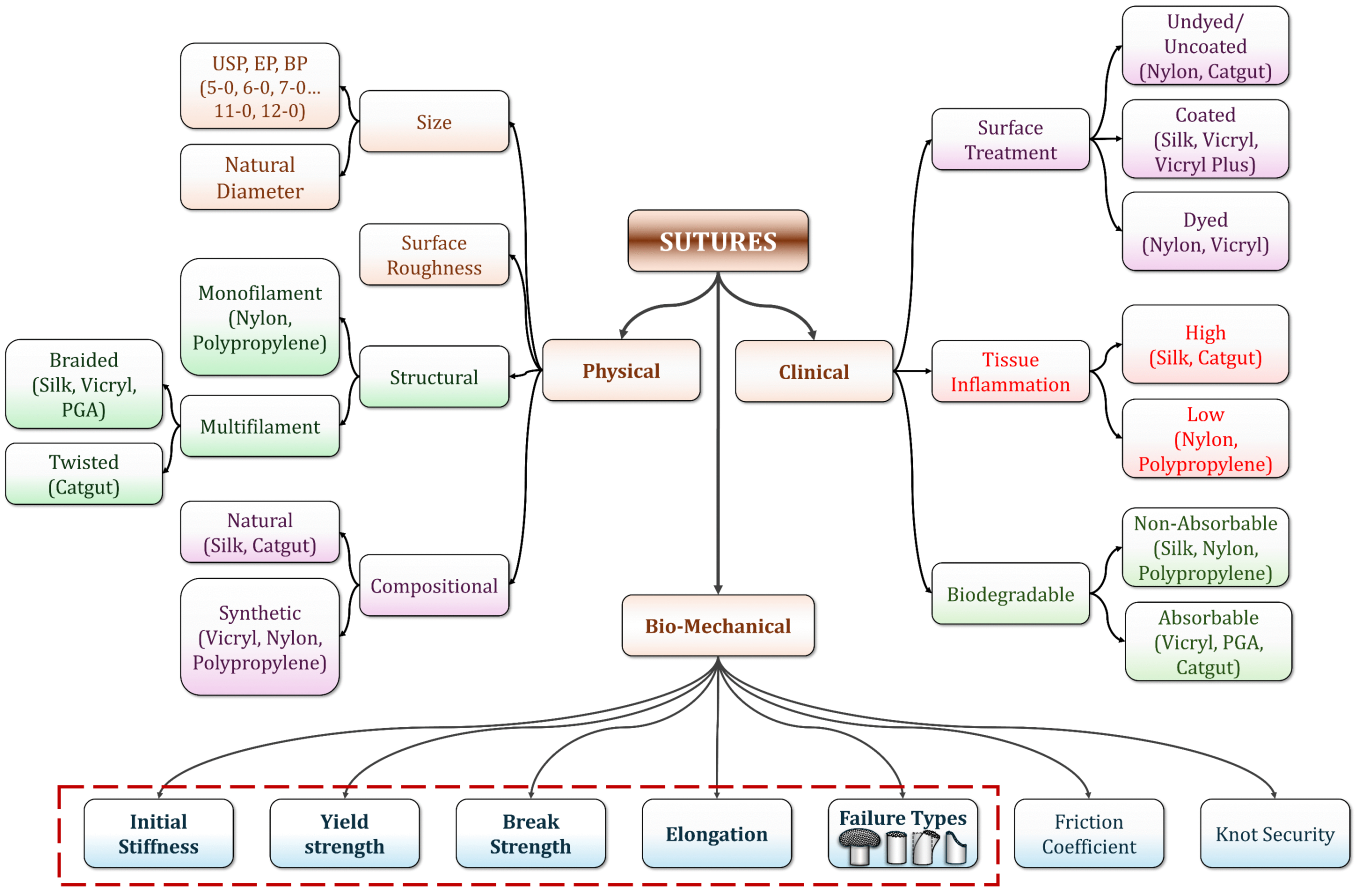
Classification of the suture materials [12,13,15,17,21].

Sutures are broadly categorized based on (i) physical, (ii) clinical, and (iii) biomechanical performance [11], as shown in Fig 1. From the physical perspective, the sutures are further classified depending on their (a) size, (b) surface roughness, (c) structural, and (d) compositional characteristics. Sutures are graded using United States Pharmacopeia (USP), European Pharmacopoeia (EP), and British Pharmacopoeia (BP) nomenclature [12]. However, USP remains the most frequently used nomenclature. As per the USP nomenclature, sutures are grouped into sizes 10–1, 0, 00(2−0), to 12−0 with decreasing gauge diameters [13,14]. Sutures are classified based on surface features as smooth or rough sutures. The smoother sutures exhibit less drag on the tissue, which is preferred as it causes less tearing of delicate tissues [15,16].

Sutures are structurally classified as monofilament or multifilament, with the multifilament sutures being either braided or twisted [17]. Multifilament sutures possess less memory and higher knot security than monofilament sutures, making them easy for surgeons to handle [16]. Whereas monofilament sutures exhibit a lower risk of inflammatory reactions, reduced capillarity, and minimal friction, which helps ease tissue passage [18]. Sutures are grouped as natural or synthetic based on their composition [19]. Natural sutures, such as silk and catgut, have limitations due to their tendency to provoke high tissue reactions and less predictable degradation rates. At the same time, synthetic sutures provide better control over healing time, reduce tissue inflammation, and lower the risk of infection [20].

Clinically, sutures are categorized based on their (a) biodegradability, (b) tissue inflammation, and (c) surface treatment characteristics [22,23]. Sutures are either absorbable or non-absorbable, based on their biodegradability. The absorbable sutures are preferred for internal suturing [24,25], and the non-absorbable sutures are preferred where the suture’s tensile strength is essential (desired) over an extended period [26]. Sutures can trigger tissue inflammation depending on the suture material and suture structure [27]. Synthetic sutures and monofilament sutures generally induce less tissue inflammation than natural sutures and multifilament sutures [28]. Sutures are classified into dyed, coated, and undyed/uncoated based on their surface treatment. Dyed sutures are easier to identify and exhibit improved suture visibility than undyed sutures [29]. Coated sutures exhibit enhanced surface properties, including smoothness, biocompatibility, reduced tissue reaction, low wettability, ease of handling, and enhanced knot security, compared to uncoated sutures [12].

The biomechanical properties of sutures play a pivotal role in load transfer, wound apposition, and preventing dehiscence. Suture mechanical failure directly increases the risk of inflammation and/or infection in the underlying tissues, leading to undesirable postoperative outcomes [30]. The major biomechanical properties of the sutures are initial stiffness, yield strength, breaking strength, elongation at break, friction coefficient, failure modes, knot strength, and knot security [31,32]. The essential biomechanical properties of the sutures in closing the soft tissue are quite different from those of hard tissues. For soft tissues, the sutures should have (i) high breaking strength to withstand the tensile load during suturing, (ii) low initial stiffness and high elongation to avoid cutting into the tissue during swelling, (iii) lower friction to decrease the tissue injury, and (iv) better knot security to protect from tissue failure and wound reopening [5,33]. Whereas for the hard tissue, the sutures should have (a) higher breaking strength to resist rupture, (b) lower elasticity to prevent excessive stretch, and (c) excellent knot security to prevent suture failure [34].

The mechanical behavior of surgical suture sizes ranging from 1−0 to 5−0 has been extensively studied in the literature [30–32,35–41]. These studies have highlighted the influence of the suture material, size, structure, knot configuration, and test conditions on the mechanical performance of the sutures [30,37–40,42,43]. However, comparatively fewer studies have reported on the mechanical characterization of low gauge sizes (6−0 to 10−0) [3,44–48]. Low-gauge diameter sutures are widely used for skin closure, facial muscle repair, and microsurgery, including ophthalmology, microvascular, and nerve procedures [4–10,49]. The mechanical properties of sutures ensure the prevention of dehiscence and proper tissue approximation, which leads to an enhanced healing process [50]. The suture strength and its elastic properties decrease nonlinearly with excessive axial twisting of sutures, leading to early suture failure and, thereby, postoperative wound complications [51].

Under surgical conditions, factors such as knot administration, knot configuration, and wetting play a noticeable role in the outcomes of wound closure. The failure load of sutures depends on the suturing techniques adopted by the surgeon [38,52]. Continuous suturing and repeated handling with instruments can cause mechanical damage, leading to serious clinical consequences, like the premature breakdown of sutures [53]. The knot resistance preserves the stability of a knot by preventing breakage and slippage under applied tension [54]. The tensile strength of the sutures in different simulated soaked conditions typically exhibits lower strength than in dry conditions [55–58]. The mechanical properties of sutures under surgical scenarios greatly depend on their in vitro mechanical properties under pristine conditions. However, under in vitro conditions, particularly in the ocular environment, the performance of sutures remains a topic of ongoing research.

The present work enhances surgical outcomes and patient care in ophthalmology by providing a profound understanding of the mechanical behavior of various sutures, including their failure morphology. In turn, these findings help in three major contributions: (i) provide the optimal suture selection for any ophthalmic surgeries, (ii) provide the required mechanical properties for any ophthalmic surgical simulations, and (iii) assist in future suture designs. The structure of this article is as follows: Section 1 illustrates the importance of sutures and their mechanical properties under surgical scenarios. Section 2 furnishes the suture materials, test configurations, and the experimental setup to determine the mechanical properties. Section 3 presents the tensile behavior and fracture surface morphology of the sutures under various test configurations. Lastly, the key findings of the study are summarized in Section 4.

## 2. Materials and experimental methods

The approvals and ethics statement are illustrated in section 2.1. The material details of the ophthalmic sutures are presented in Section 2.2. The experimental configurations of the sutures under tensile loading are presented in Section 2.3. The details regarding SEM fractography and statistical analysis are presented in Sections 2.4 and 2.5, respectively.

### 2.1. Approvals and ethics statement

This study was carried out with the protocols approved by (i) the Ethics committee of the Indian Institute of Technology Hyderabad (protocol: IITH/IEC/2024/01/[03 and 04]), and (ii) the L V Prasad Eye Institution review board (protocol: LEC-BHR-P-12-23-1151). Furthermore, this study did not involve any investigations involving human participants, human or animal tissues, vertebrate animals, embryos, or field research. All tests in the present investigation were performed, and the corresponding experimental data were collected between 17/03/2024 and 11/11/2024.

### 2.2. Suture materials

Sutures of size 6−0 to 10−0, comprised of Vicryl, Nylon, and Polypropylene, are often used for Ophthalmic surgical applications. Table 2 lists the properties of various suture types tested in the present study [14,59]. The Vicryl sutures (M/s. Ethicon, India) and the Nylon and Polypropylene sutures (M/s. Aurolab, India) are used in the present study. The Vicryl suture is a synthetic, braided suture. It is composed of Polyglactin 910, a copolymer of 90% glycolic acid and 10% lactic acid. The Nylon and Polypropylene sutures are synthetic and monofilament. Nylon is formed of long-chain aliphatic polymers made from 100% homopolymers of Nylon 6 and Nylon 6.6. While Polypropylene is composed of an isotactic crystalline stereoisomer. In the current investigation, the uniaxial tensile properties were examined for (i) Vicryl sutures 6–0/7–0/8–0, (ii) Nylon sutures 8–0/9–0/10–0, and (iii) Polypropylene sutures 9–0/10–0.

**Table 2:** Specifications of sutures investigated for mechanical properties.

Type	Composition	Size	Gauge no.	Structure	Absorbable	Model
**6−0 Vicryl**	Polyglactin 910	6−0	0.7	Multi/Braided	Yes	NW2670
**7−0 Vicryl**		7−0	0.5			NW9561
**8−0 Vicryl**		8−0	0.4			NW2301
**8−0 Nylon**	Nylon 6, 6.6	8−0	0.4	Mono	No	8482N
**9−0 Nylon**		9−0	0.3			6492N
**10−0 Nylon**		10−0	0.2			6492N
**9−0 Polypropylene**	Polypropylene	9−0	0.3	Mono	No	60692PP
**10−0 Polypropylene**		10−0	0.2			60602PP

Each suture type listed in Table 2 is tested in three configurations: straight configuration (SC), knotted configuration (KC), and looped configuration (LC), with five samples for each test configuration. The details of various test configurations explored in the present study are described in Section 2.2. All sutures were visually inspected for damage like frays, kinks, breaks, and contamination. Damage-free sutures, within their expiration date, were used in the experiments.

### 2.3. Test Configurations: Straight, Knotted, and Looped

In the present study, the mechanical properties of the suture were investigated under SC, KC, and LC conditions. The uniaxial testing of the above three suture configurations was performed using a universal testing machine (UTM) (Model 5944, Instron). Typically, the suture load-carrying capacities of 6−0 to 10−0 sutures vary from ~1 to 12N. Hence, the Instron 5944 inbuilt load cell was swapped with the 5/10/50N load cell (Model 2530-5N/10N/50N, Instron), with an accurate measurement of $\frac{\text{1}}{\text{5}\text{0}\text{0}}$^th^ of the load cell capacity for testing sutures.

**Fig 2:**
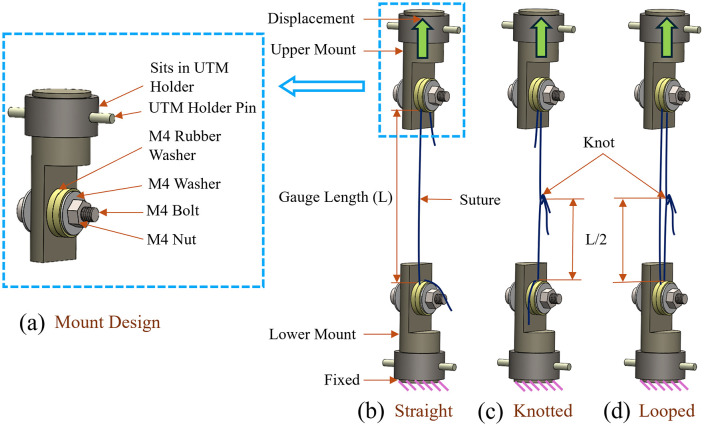
(a) The schematic representation of the modified custom-designed mount to grip the suture, experimental setup for (b) straight, (c) knotted, and (d) looped configurations.

The most challenging aspect during the uniaxial testing of the suture is its slippage at (i) the mount ends and (ii) the knots. The suture slippage at the mount ends was prevented in the present experiments through a custom-designed mount, as shown in Fig 2a. The mount employed in the earlier work of authors to study the mechanical strength of the corneal graft-host-junction was further modified with rubber washers to grip the suture firmly [60]. The mount contains (a) UTM holder pin for alignment and fixation with the load cell, (b) two rubber washers to ensure proper griping of the sutures, (c) two mild steel washers to prevent the bolt head and nut from digging in, (d) a bolt to hold the suture in place, and (e) a nut to properly tightening the rubber washers. The suture specimens were loaded in SC, KC, and LC, as shown in Figs 2b-d, respectively.

**Fig 3:**
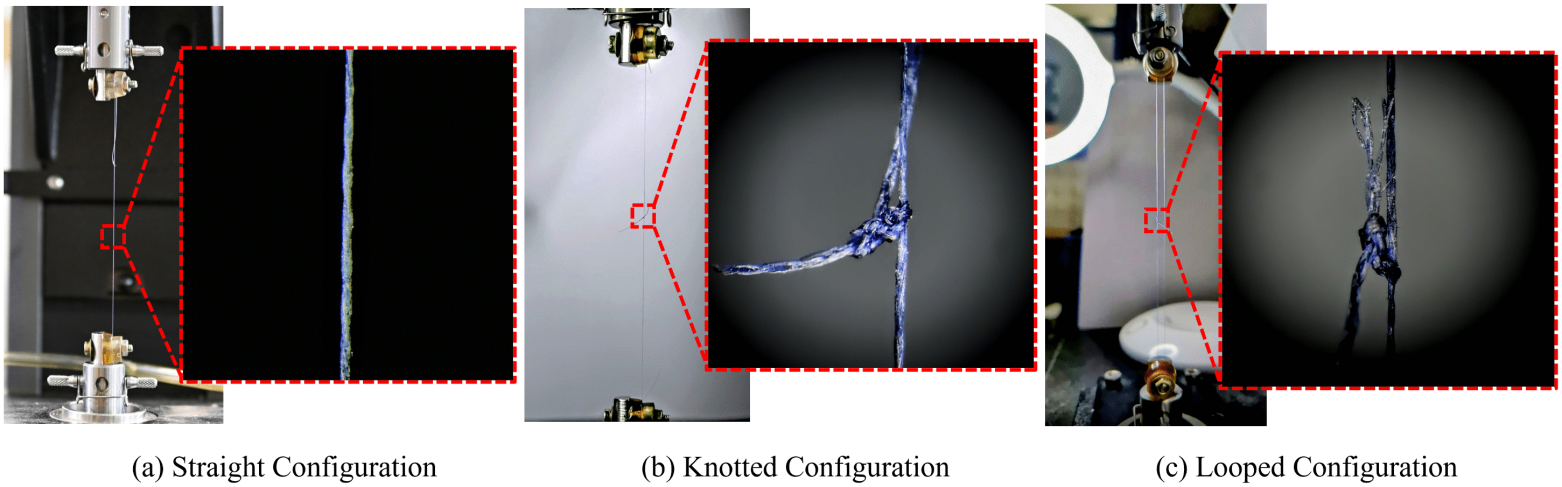
The experimental snapshots for (a) straight, (b) knotted, and (c) looped configurations.

The sutures were loaded to the mount for the SC by wrapping the sutures on the M4 bolt, as shown in Fig 2b. The suture was gripped between two rubber washers to ensure the uniform gripping of the suture with the M4 bolt. Fig 3a shows the suture specimen gripped on a customized mount in the SC. The KC illustrated in Fig 2c was obtained by forming a loop of suture over the M4 bolts and knotting the free ends. Then, the suture strand without a knot is cut, and the cut ends are gripped firmly to the M4 bolts on the clamp using rubber washers. For the LC, the sutures were loaded to the mount by the loop produced by the knot on the suture over the M4 bolt, as shown in Fig 2d. For the KC and LC, the sutures were aligned so that the knot was in the middle of the gauge length.

**Fig 4:**
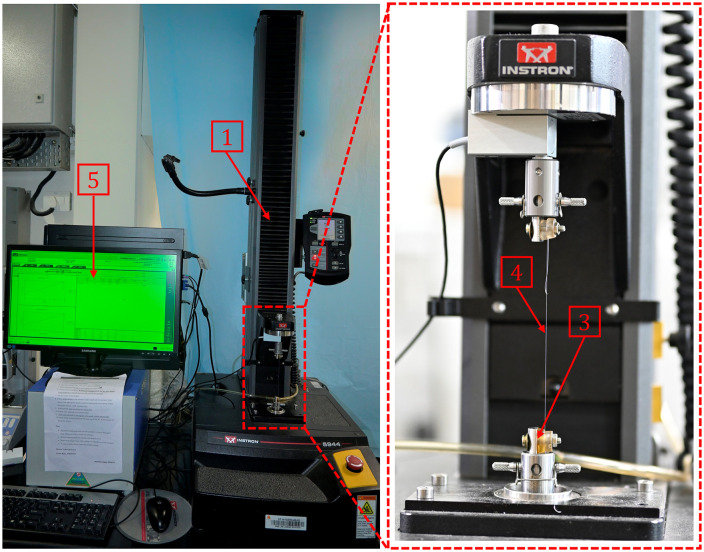
The Experimental setup for evaluating the mechanical properties of the sutures contains 1 the Uniaxial Testing Machine (UTM), 2 the Load Cell, 3 custom-designed mount to firmly grip 4 the suture, 5 data acquisition system and recording device (not shown in the image).

The sutures were knotted using square knots with four throws for both KC and LC [54]. The Fig 3b and c illustrate the suture specimen mounted in KC and LC with a high-magnification image showing knot location and configuration. The cut ends of the knots were maintained at more than 5 mm to avoid slippage and untying of the knot [30,61]. Knotting of the sutures was carried out in-house after getting proper training from surgeons. The nomenclature of the suture specimens tested in the present study is as [*n-0*][V/N/P][S/K/L][#]. Where *n-0* represents the size of the sutures, V/N/P represents Vicryl/Nylon/Polypropylene material type, S/K/L represents straight/knotted/looped configuration for tensile test, and # represents the specimen number. The experimental setup used to evaluate the mechanical properties of the sutures is shown in Fig 4. It includes a UTM equipped with a 5/10/50N load cell, customized mounts to hold the suture in place securely, a DAC card to acquire test data, and a computer to operate the UTM.

The gauge length for the sutures in all three configurations was kept constant at 120 mm, based on the lower limit achievable in the UTM setup. For each experiment, the gauge length and location of the knot at the center were ensured using vernier calipers. The experiments were carried out at a strain rate of 30 mm/min in the present study. It results in a testing time of around 20–90 seconds, which is closer to the ASTM D2256/D2256M-21 recommendation of 20 ± 3 seconds test time. Unlike the low strain rate (3 mm/min), as reported in the literature, which yields a test time of 120–180 seconds [44,46,47].

### 2.4. Fractography analysis

High-magnification fractography characterization was conducted using a Zeiss EVO18 Scanning Electron Microscope (SEM) to investigate the fracture behavior of the remnant sutures from the uniaxial tension test. The remnant sutures were prepared for SEM imaging by sputtering with a thin layer of gold coating using a vacuum sputter (Quorum SC7620 Sputter Coater). SEM imaging was carried out at an accelerating voltage of 10 kV. The SEM fractography characteristics were compared with the mechanical testing results to study the failure mechanisms of sutures under tensile loading conditions. Fracture morphology and failure behavior of sutures were investigated for SC, KC, and LC, and presented in section 3.2

### 2.5. Statistical investigation

A descriptive statistical investigation was conducted to comprehensively examine the mechanical characteristics of each suture type. A one-way ANOVA test was employed to assess the statistically significant differences in mechanical properties with respect to suture types and test configurations. The statistical significance of the suture’s mechanical properties, such as the initial stiffness, yield strength, breaking strength, and elongation at break, was evaluated for the SC, KC, and LC, with a significance level of 0.05. The experimental results for the mechanical properties of the sutures were presented as mean ± standard deviation. Tukey’s post hoc test was used to identify specific group differences. In the present work, Origin 2024b was used to perform statistical analyses and visualize the results.

## 3. Results and discussion

The mechanical characteristics of sutures are presented in Section 3.1. The fracture surface morphology is discussed in Section 3.2. Statistical significance and the surgical relevance of the mechanical properties are demonstrated in sections 3.3 and 3.4, respectively.

### 3.1. Mechanical characterization of sutures in straight, knotted, and looped configurations

**Fig 5:**
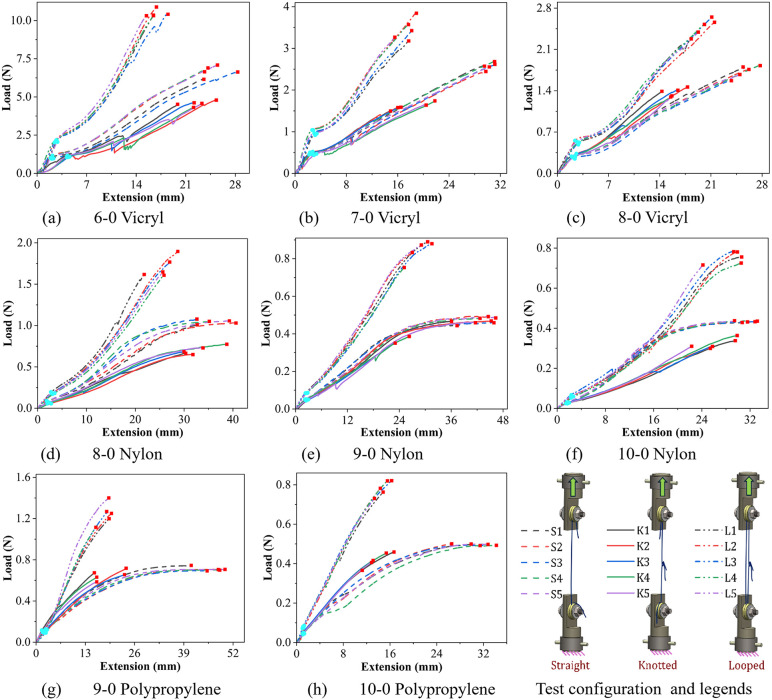
The load vs. extension curve in straight, knotted, and looped configurations of (a)-(c) 6-0/7-0/8-0 Vicryl, (d)-(f) 8-0/9-0/10-0 Nylon, (g)-(h) 9-0/10-0 Polypropylene.

The deformation behavior of sutures is of fundamental interest in understanding their performance in surgical scenarios. Figs 5a-c illustrate the load vs. extension curves of 6–0/7–0/8–0 Vicryl, Figs 5d-f illustrate the load vs. extension curves of 8–0/9–0/10–0 Nylon, and Figs 5g-h illustrate the load vs. extension curves of 9–0/10–0 Polypropylene, under SC, KC, and LC. From the uniaxial tests, initial stiffness, yield strength, break strength, and elongation at break were probed. The load vs. extension curves obtained in (i) the SC are presented in dashed lines, (ii) the KC are presented in solid lines, and (iii) the LC are presented in dash-dot-dot lines. Key points, such as the yield point and the breaking point of the suture in the load vs. extension curve, are represented by cyan dots and dark red squares, respectively, for easy identification. Vicryl and Nylon sutures in all test configurations exhibit an *S*-shaped load vs. extension response (See Figs 5a-c and 5d-f), while the Polypropylene sutures exhibit an *R*-shaped response (see Figs 5g-h). The raw data that generates the load vs. extension curves is provided in the supplementary S4 Data.

The key features of the *S*-shaped (sigmoidal shape) load vs. extension curve obtained from the uniaxial tensile test of sutures are as follows. The initial small curved region encountered at the beginning of the load vs. extension curve is the crimp region [36]. The braided geometry, such as the Vicryl sutures, exhibits this distinctive trait of crimp. At low loading, filaments of braided suture reorient (filament-sliding takes place), and the braid angle decreases [62]. By increasing the tension, the braided structure begins to reach a jammed state, and the mechanical response becomes dependent on the filament properties once the braid structure is fully jammed [63]. The crimp region is followed by a linear region, indicating the linear elastic deformation. The linear region continues till the yield point. Beyond the yield point, the suture deforms plastically. Post yield, the suture continues to deform with a low increase in load. This region is the linear strain-hardening region. Subsequently, the curve ascends exponentially, indicating a rapid strain hardening region. Eventually, the load vs. extension curve saturates at some load level and continues to elongate till the breaking point, where the suture ruptures. The yield point determines the yield strength, and the breaking point determines the breaking strength and the elongation at break. Meanwhile, the key features of the *R*-shaped and S-shaped load vs. extension curves are similar. However, the crimping region and the exponential strain hardening region are absent in the *R*-shaped curve.

Fig 5a represents the load vs. extension response of 6−0 Vicryl sutures tested in SC, KC, and LC. The SC exhibited a smooth S-shaped curve, whereas the KC and LC showed a slight drop in load in the inelastic region. It is due to a sudden decrease in the load-carrying capacity once the knot gets fully developed [64]. From Fig 5a, it is observed that the yield strength, break strength, and elongation at break for the SC are consistently higher than those of the KC. In contrast, the yield strain is lower for the SC, which is due to the difficulty in ensuring proper knot tightening of sutures in KC for the thick multifilament suture (6−0 Vicryl). The reduction in yield strength, break strength, and elongation at break for the KC is attributed to (i) stress concentrations near the knot location, (ii) the orientation of force at an acute angle to the suture axis, and (iii) increased friction between filaments within the knot [31,63,64]. Supplementary video S1 File shows the deformation of a 7−0 Vicryl under a looped configuration until fracture.

The load vs. extension behavior of the LC shown in Fig 5a can be approximated as a combination of one SC and KC loaded in parallel (under displacement control). Hence, the yield strength in the LC is the sum of the SC and KC, as the deformation is linear and results in the linear superposition. The break strength is approximately the sum of the straight and knotted strands, as the deformation is non-linear at the breaking point. The elongation at break for the LC must be the lowest of the SC and KC, as the loading is through displacement control. The KC elongation at break is the lowest; hence, the LC elongation at break is approximately equal to the KC. For all the LCs presented in Figs 5a-h, the above approximation with the corresponding suture’s SC and KC is valid.

Overall, for the 6−0 Vicryl sutures, the yield strength, initial stiffness, and break strength are highest in the LC, followed by the SC and KC. The elongation at break is maximum in the SC, followed by KC and LC. Figs 5b-c illustrates the load vs. extension response of 7–0/8–0 Vicryl sutures tested in the SC, KC, and LC. The yield strength, break strength, and elongation at break of 7–0/8–0 Vicryl sutures show a similar trend to 6−0 Vicryl sutures. However, a 7–0/8–0 gauge diameter is less than a 6−0 gauge. Hence, it resulted in two significant changes for the KCs: (i) a decline in elongation at break and (ii) a decrease in sudden load drop due to the knot with the decrease in gauge diameter. Further, the overall strain hardening for the KC over SC was lower for 6−0, similar for 7−0, and higher for the 8−0 Vicryl.

Figs 5d-f presents the load vs. extension behavior of 8–0/9–0/10–0 Nylon sutures in SC, KC, and LC. They exhibit an *S*-shaped load vs. extension curve, similar to Vicryl sutures. However, the two major differences observed in Nylon sutures are (i) they do not have a crimp region, as Nylon sutures are monofilament polymers in comparison to multifilament Vicryl, and (ii) the sutures tested in SC and KC exhibited a larger saturation region before failure. The overall trend in yield and breaking strength of the Nylon sutures is similar to that of Vicryl sutures. While the elongation at break for Nylon sutures among all test configurations increased from 8−0 to 9−0 sutures and then decreased from 9−0 to 10−0 sutures. Further, the overall strain hardening for the KC w.r.t the SC was lower for 8−0 and 10−0, but similar for 9−0 Nylon Sutures, which can be attributed to the size effects of the knot and the uncertainty in the knot administration with lower gauge diameters on the overall mechanical performance. Supplementary video S2 File provides the deformation of a 10−0 Nylon under knotted configuration till fracture.

Figs 5g-h presents the load vs. extension behavior of 9–0/10–0 Polypropylene sutures in SC, KC, and LC. They exhibit an *R*-shaped load vs. extension response, unlike the S-shaped behavior exhibited by Vicryl/Nylon. In comparison with Nylon sutures, the Polypropylene sutures exhibited a higher value of initial stiffness and break strength for the corresponding gauge diameter. Meanwhile, the elongation at break of Polypropylene was comparable to Nylon in the SC. However, in the presence of knots (KC and LC), the Polypropylene sutures failed far earlier than the Nylon sutures. Supplementary video S3 File provides the deformation of a 10−0 Polypropylene under knotted configuration till fracture. To summarise, this section presented the qualitative response of sutures under uniaxial tension. A detailed quantitative comparison of their mechanical behavior is presented in Section 3.3.

### 3.2. Fractography

The SEM studies were conducted on remnant sutures to capture the fracture morphologies and mechanisms under uniaxial tensile loading for SC, KC, and LC. The fracture mechanisms in the sutures are classified based on the loading into the following: (1) tensile failure, (2) tensile-shear failure, and (3) brittle failure [65]. The tensile failures are further combined (i) with/without plastic deformation (PD) and (ii) with/without adiabatic heating. During the tensile failure, the crack initiates in the fiber either from points/lines/arcs on the surface or internal flaws. Excessive elongation of the suture results in V-notch formation during the crack propagation stage [66]. Subsequently, a catastrophic failure occurs due to the greater stress in the fiber. Meanwhile, the presence of plastic deformation causes permanent deformation of the sutures, resulting in a significant permanent set that leads to curvature near the fracture in the otherwise straight fibers [67]. Typically, the knots in the sutures cause lateral loading, resulting in a low- or high-stress concentration in the vicinity of the knots due to combined tensile-shear loading. This behavior is observed both in the KC and LC. The fracture in these configurations almost occurs near the knot location. Further, the lack of quick heat dissipation to the environment causes the melting and fusion of the fiber breaks.

**Fig 6:**
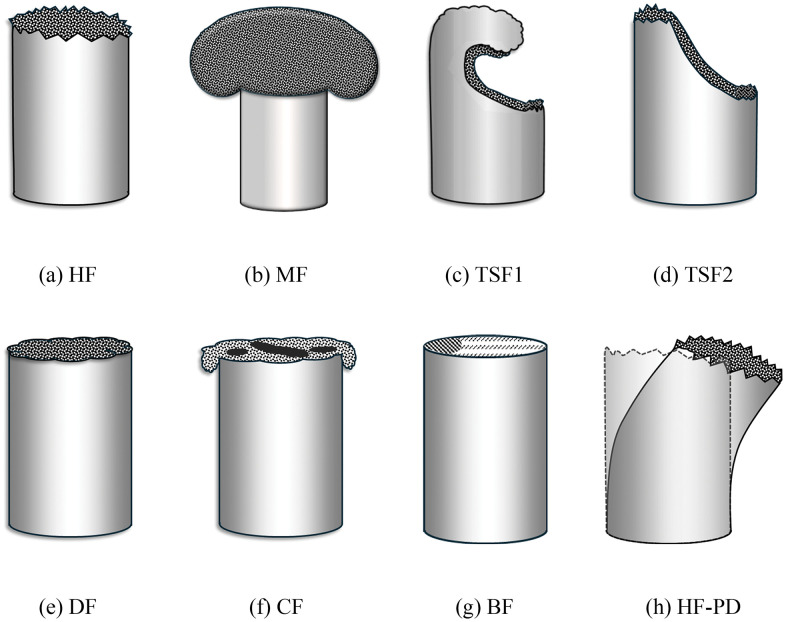
Failure modes of fibers: (a) Heakled failure (HF), (b) Mushroom failure (MF), (c) Tensile-Shear failure-1 (TSF-1), (d) Tensile-Shear failure-2 (TSF-2), (e) Distributed failure (DF), (f) Confusion failure (CF), (g) Brittle failure (BF), and (h) Hackled failure with permanent deformation (HF-PD).

Based on the above-discussed loading scenarios, the failure modes are broadly classified into (see Fig 6): (a) Hackled failure, HF: low-strain-rate tensile break; (b) Mushroom failure, MF: high-strain-rate tensile break; (c) Tensile-Shear failure-1, TSF1: combined tensile-shear failure initiated from a single crack; (d) Tensile-Shear failure-2, TSF2: combined tensile-shear due to the coalescence of axially displaced tensile cracks followed by shear deformation; (e) Distributed Failure, DF: tensile failure with adiabatic heating and fusion; (f) Confusion Failure, CF: combined tensile-shear failure with adiabatic heating and fusion; (g) BF: Brittle failure [68]. The failure modes through (a)-(f), when assisted by the PD, cause curvature in the sutures, as shown in Fig 6h. BF morphology was seldom observed due to the ductile nature of the sutures selected for the current study.

**Fig 7:**
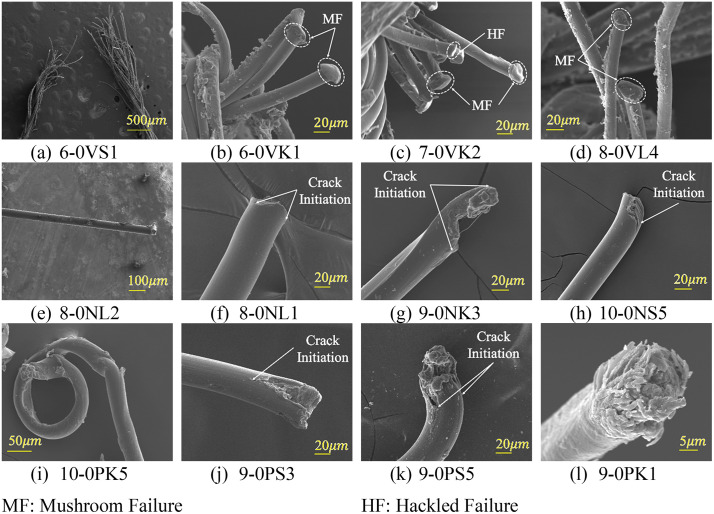
SEM fractography of (a-d) Vicryl, (e-h) Nylon, and (i-l) Polypropylene sutures failed under uniaxial tensile load.

Figs 7a-d presents the fracture morphology of 6–0/7–0/8–0 Vicryl sutures. Fig 7a shows the multiple fractured fibrils of the 6−0 Vicryl suture at lower magnification, showcasing the overall fracture of multifilament braided Vicryl. Figs 7b-d shows the independent fibrils’ fracture morphology at greater magnification for the 6–0/7–0/8–0 Vicryl, respectively. It was observed that the fibrils of Vicryl sutures mainly exhibited MF, followed by HF morphology, as presented in Table 3. During high-speed tensile failure (MF), the initial crack geometry evolves into a mushroom shape due to the combined effect of snapback and the accumulation of adiabatic heat. Meanwhile, for the lower-speed tensile failure (HF), the crack surface remained flat and roughened. Occasionally, some fibrils of multifilament Vicryl from KC and LC exhibited TSF1 morphology.

**Table 3:** Frequently observed fracture morphologies in Ophthalmic sutures.

Types of Sutures	Configurations		
	Straight	Knotted	Looped
6−0 Vicryl			
7−0 Vicryl			
8−0 Vicryl			
8−0 Nylon			
9−0 Nylon			
10−0 Nylon			
9−0 Polypropylene			
10−0 Polypropylene			

Figs 7e-h presents the fracture morphology of 8–0/9–0/10–0 Nylon sutures. Fig 7e shows the monofilament 8−0 Nylon suture after fracture at lower magnification. Fig 7f illustrates the HF morphology obtained due to multiple crack initiation lines in the 8−0NL1 sample. Fig 7g illustrates the TSF2 morphology obtained due to multiple cracks that are axially displaced, followed by excessive shear deformation, in the 9−0NK3 sample. Fig 7h illustrates the HF morphology obtained due to crack initiation from an arc on the surface of the 10−0NS5 sample. Overall, in 8–0/9–0/10–0 Nylon sutures, majorly HF morphology was observed in all configurations, while a few fibers exhibited TSF2 morphology for KC and LC, as listed in Table 3.

Figs 7i-l presents the fracture morphology of 9–0/10–0 Polypropylene sutures. Fig 7i illustrates an LC of a monofilament 10−0 Polypropylene suture failed at the knot with TSF2-PD failure morphology. Fig 7j illustrates the HF morphology obtained due to crack initiation from a point on the 9−0PS3 sample. Fig 7k illustrates the DF(-PD) morphology obtained due to tensile failure from multiple crack initiation, followed by excessive adiabatic heating. Further, it also exhibited slight PD. Fig 7l illustrates the CF(-PD) morphology obtained due to tensile-shear failure, followed by adiabatic heating in the 9−0PK1 sample. Due to excessive localized melting and fusion with elongation on the fracture surface, the original crack initiation and stable crack regions were not seen in the CF morphology, as shown in Fig 7l. Overall, 9–0/10–0 Polypropylene sutures exhibited similar failure morphology to Nylon sutures. However, two major differences between Polypropylene sutures in comparison to Nylon are: (i) the majority of fibers exhibited TSF2 failure over HF, as shown in Table 3, and (ii) a more irregular fracture surface was seen for the Polypropylene sutures due to their peculiar chemical and thermomechanical behavior.

### 3.3. Statistical investigation

**Fig 8:**
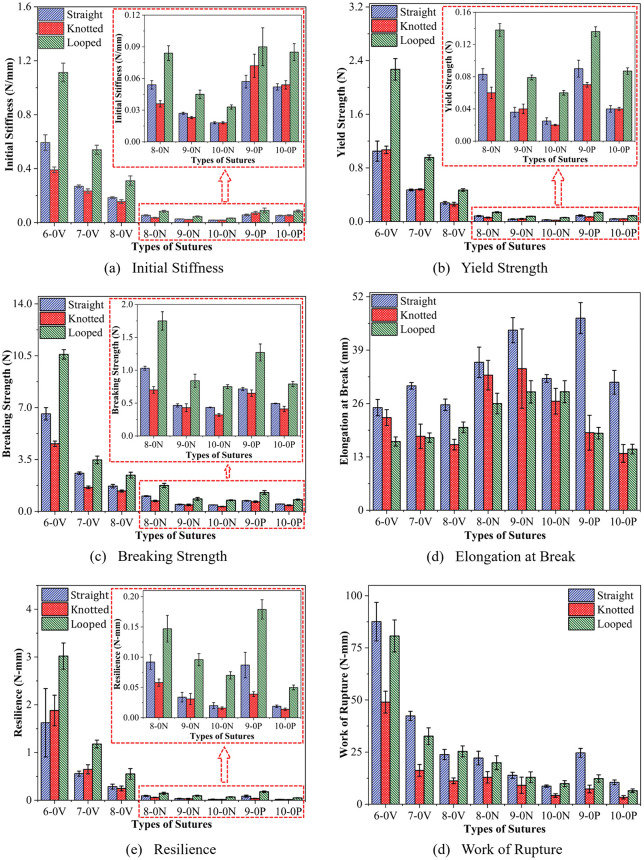
(a) Initial stiffness, (b) yield strength, (c) breaking strength, (d) elongation at break, (e) resilience, and (f) work of rupture for 6–0/7–0/8–0 Vicryl, 8–0/9–0/10–0 Nylon, 9–0/10–0 Polypropylene sutures in straight, knotted, and looped configurations.


The statistical variation obtained from the initial stiffness, yield strength, breaking strength, elongation at break, resilience, and work of rupture is summarized in Figs 8a-f. The values of yield strength, breaking strength, and elongation at break were obtained from load vs. extension data. The horizontal axis of the load vs. extension curve denotes elongation “x” and the vertical axis presents the load “F(x)”. The initial stiffness, resilience, and the work of rupture were calculated using the load vs. extension data and the suture’s geometry. The initial stiffness was determined from the slope of the linear region of the load vs. extension curve. Whereas the resilience and work of rupture were obtained by integrating the load vs. extension curve up to the elastic limit and the point of failure, respectively [69].

$$\begin{array}{c} \text{Initial}\;\text{Stiffness}\;(k)=\frac{\Delta \text{F}}{\Delta x}=\frac{F_{e}-F_{\text{0}}}{x_{e}-x_{\text{0}}} \end{array}$$ (1)

$$\begin{array}{c} \text{Resilience}\;(U)=\;\int_{x_{\text{0}}}^{x_{e}}F(x)dx \end{array}$$ (2)

$$\begin{array}{c} \text{Work}\;\text{of}\;\text{Rupture}\;(W)=\;\int_{x_{\text{0}}}^{x_{f}}F(x)dx \end{array}$$ (3)

where $F_{\text{0}}\text{ and }F_{e}$ shows the load at the initial and elastic points respectively. The elongation at the initial, elastic, and failure points is represented by $x_{\text{0}},\;x_{e},\text{ and }x_{f}$ respectively. F(x) denotes the load at the elongation x.

Fig 8 summarizes the influence of gauge diameter and material on the tensile behavior of surgical sutures. Across all materials and configurations, a decrease in initial stiffness, yield strength, breaking strength, resilience, and work of rupture was observed as the diameter decreased (Figs 8a–c). [37]. Similar diameter-dependent reductions have been reported for Nylon, Polypropylene, and Vicryl sutures under tensile loading [36–38].

Among all tested sutures, the 6−0 Vicryl sutures tested in LC exhibited the highest initial stiffness (1.11 ± 0.07 N/mm), yield strength (2.29 ± 1.12 N), break strength (10.45 ± 0.26 N), and resilience (3.02 ± 0.27 N-mm) due to their higher gauge diameter and twice the strands as SC and KC. The tensile strength of 6−0 Vicryl suture in SC is 6.67 ± 0.32 N, where a similar value was reported by Brooks et al as 6.1 N (623.5 g) [48]. The tensile strength in KC is typically 20–50% lower than that in SC, depending on the polymer type, filament architecture, and gauge [31]. Calfeeon et al. demonstrated that looped and multi-strand sutures show comparable increases in stiffness and peak load relative to single-strand constructs [39]. The breaking strength in SC decreases by 61% between 6−0 and 7−0 Vicryl suture, whereas it shows a 34% drop between 7−0 and 8−0 Vicryl sutures. Brauner et al. reported the 37% drop in breaking strength between 7−0 and 8−0 Vicryl sutures [44]. Similarly, the strength drops by 54%, 7%, and 31% between 8–0/9–0 Nylon, 9–0/10–0 Nylon, and 9–0/10–0 Polypropylene sutures, respectively.

The 10−0 Nylon sutures tested in the KC exhibited the lowest initial stiffness (0.017 ± 0.001 N/mm), yield strength (0.022 ± 0.001 N), and breaking strength (0.324 ± 0.025 N), shown in Figs 8a–b. This behavior reflects the combined influence of small suture diameter and stress concentration at the knot, where bending, frictional sliding, and localized damage accelerate crack initiation. In comparison, the 10−0 Polypropylene sutures tested in KC had lower resilience (0.014 ± 0.002 N-mm) and lower work of rupture (3.324 ± 0.803 N-mm) than 10−0 Nylon sutures due to their lower elongation at break and early failure due to the knot. Similar observations have been reported by Greenwald et al. and Wang et al., who demonstrated that sutures in SC consistently exhibit greater failure load than sutures in KC [40,42]. The reduction in tensile strength of knotted sutures typically ranges from 20% to 50%, depending on polymer type and gauge diameter. These findings align with previous experimental studies by Naleway et al. and Wang et al. [31,42]. Although Nylon retained moderate energy absorption due to its relatively high ductility, the 10−0 Polypropylene sutures tested in KC showed the lowest resilience and work of rupture (Fig 8c), indicating premature failure under localized loading. This finding is consistent with previous reports describing Polypropylene as having inferior knot toughness and lower energy absorption compared to Nylon [41,43,70–72].

The elongation at break showed a different trend compared to other mechanical properties (Fig 8d). The sutures tested in the SC exhibited the highest elongation at break compared to KC and LC, as the sutures in the SC lack knots to introduce stress concentration. Similar configuration-dependent elongation behavior has been reported for both monofilament and braided sutures, where the introduction of knots significantly suppresses elongation due to stress concentration and constrained filament mobility [31]. For KC and SC, elongation at break decreased with decreasing gauge diameter for Vicryl, whereas LC showed the opposite trend.

The 6–0/7–0/8–0 Vicryl sutures had exhibited relatively lower elongation at break in SC than 8–0/9–0/10–0 Nylon and 9–0/10–0 Polypropylene sutures due to the lower mobility of fibrils in the braided structure. The highest elongation at break was recorded for 9−0 Polypropylene sutures in SC (46.780 ± 3.776 mm), followed by 9−0 Nylon (43.868 ± 3.236 mm) and 8−0 Nylon (36.042 ± 3.703 mm). The high elongation at break for 9−0 Polypropylene, followed by 9−0 and 8−0 Nylon sutures in the SC show the high ductility of monofilament polymers under uniform tensile loading. Conversely, the lowest elongation at break was observed for 10−0 Polypropylene sutures in KC, highlighting the susceptibility of fine monofilament sutures to knot-induced embrittlement. Overall, Fig 8d shows that elongation at break was lower for Polypropylene sutures in both KC and LC than for Nylon and Vicryl. For 9−0 Polypropylene, elongation in KC and LC decreased to approximately 40% and 43% of the SC value, respectively. For 10−0 Polypropylene, the corresponding reductions were approximately 44% and 47%. These reductions are in close agreement with previously reported losses in extensibility for knotted Polypropylene sutures [43,70,72,73]. The lower elongation in KC and LC for polypropylene sutures may be due to the high stress-concentration caused by multiaxial loading at the knot location.

A one-way ANOVA was performed to evaluate the statistical significance of variations in the mechanical properties of all tested sutures in SC, KC, and LC. The analysis revealed that the magnitude of the mechanical properties of the sutures varied significantly across all test configurations, with p-values less than the significance level (α = 0.05). However, the work of rupture for 9−0 Nylon (p = 0.076) and elongation at break for 10−0 Nylon (p = 0.230) showed a similar range of values in SC, KC, and LC, confirming the null hypothesis from the one-way ANOVA. Upon close observation, it can be attributed to a type-2 error (false negative). The elongation at break is a direct measure of ductility. Likewise, the work of rupture is an indirect measure of ductility, and it is directly proportional to the product of break strength and elongation at break. A huge variation in the knot administration resulted in a noticeable variation in the elongation at break in the KC and LC (see Fig 8d). However, it showed profound implications for the elongation at break for 9−0 (p = 0.038)/10−0 (p = 0.230) Nylon sutures and work of rupture for 9−0 (p = 0.076) Nylon, confirming the type-2 error. The variation in knot administration calls for a larger sample set.

Tukey’s post hoc test was performed to examine the significant variations in the mechanical properties of the sutures across different configurations. Most sutures, including 7–0/8–0 Vicryl, 10−0 Nylon, and 9–0/10–0 Polypropylene, exhibited similar initial stiffness in SC and KC. Likewise, 6–0/7–0/8–0 Vicryl, 9–0/10–0 Nylon, and 10−0 Polypropylene showed similar yield strength and resilience in SC and KC. The break strength of Vicryl, Nylon, and Polypropylene sutures varied considerably in all pairs of configurations except 9−0 Nylon and 9−0 Polypropylene in SC and KC. It was also observed that the work required to rupture the sutures varied significantly across all pairs of configurations, except for the 6–0/8–0 Vicryl and 8–0/9–0/10–0 Nylon sutures in SC and LC. This implies that the variation in knot administration doesn’t considerably affect elastic and early plastic properties for the majority of suture types tested.

### 3.4. Surgical relevance

Sutures with suitable mechanical properties are vital for effective surgical intervention and wound healing. The selection of sutures in surgical procedures by surgeons to date has typically been based on best practices passed down from one generation to another. The present study elucidates the scientific rationale behind the selection of appropriate sutures in ophthalmology, stemming from experimental investigations into the mechanical behavior of sutures. According to the present study, with a specific focus on Ophthalmic surgeries, Vicryl sutures are found to be preferable for stiffer tissue apposition, which requires higher stiffness and greater break strength, along with a lower elongation level. Hence, Vicryl sutures can be used for surgeries that involve tissues such as eyelid skin (6−0), extraocular muscles (7−0), Sclera, and Conjunctiva (8−0). Further, clinically, it is observed that Vicryl is more challenging to loop and tie knots compared to Nylon/Polypropylene.

In contrast, Nylon sutures are suitable for soft tissue repair, where greater flexibility and higher elongation are necessary to protect the tissue from drag or tear out due to excessive swelling or movement. The soft tissue, such as the cut edges of the cornea, loses stiffness due to swelling [74,75]. Hence, it requires sutures to achieve proper apposition and to redistribute loads effectively without causing localized stress concentrations. The enhanced ductility of Nylon sutures ensures the suture loop tightens uniformly and maintains force transmission across the apposed cut edges without premature tissue damage. They deliver superior strength across the cut edges of the tissue after applying loop knots. Therefore, Nylon sutures are best suited for limbus closure (8–0/9–0) and corneal surgeries (10−0). Likewise, Polypropylene sutures are preferable where moderate stiffness and moderate break strength with greater elongation are required, such as scleral fixations of intraocular lenses (8–0/9–0) and corneal surgeries (10−0). The tensile test results presented in Fig 8 indicate that Polypropylene sutures exhibit higher initial stiffness, greater yield strength, and superior breaking strength compared to Nylon. Owing to these properties, Polypropylene undergoes minimal deformation and does not yield under the tensile forces typically encountered during intra-operative and post-operative conditions. Its high strength and limited elongation enable the suture to maintain wound approximation without premature failure [76]. Hence, Polypropylene is commonly used in repairing minute, thinner tensile intraocular structures like the iris in an iridodialysis repair and lid suspension procedures.

The present investigation on sutures offers a comprehensive and standardized Mechanical characterization of the sutures used in ophthalmology applications (gauge sizes 6–0 to 10–0). The tests were conducted in three configurations, *i.e.*, straight (SC), knotted (KC), and looped (LC) configurations, which are important for clinical applications. In contrast to previous investigations, this study employs (a) ASTM standard (D2256/D2256M-21) for preparing the experiment protocol and performing the experiments, and (b) a custom-designed mount to mitigate the friction and stress concentration at the gripping locations [44,46–48]. High-magnification fractographic investigations were performed on the fracture surfaces of the sutures to understand the role of material- and configuration-specific fracture behavior.

A limitation of the present study is that all tests were conducted in a controlled in vitro environment (in air under dry conditions). These conditions ensure repeatability and reduce experimental variability, but do not capture the effects of fluid-mediated lubrication or frictional alterations [77,78]. Tissue-suture interaction phenomena, including tissue compliance, physiological hydration, and wound healing responses, were not incorporated. Further, the knot security, fatigue behavior, and long-term mechanical durability of the sutures are other factors that greatly influence surgical outcomes. In addition, the finite element analysis (FEM) will provide a detailed representation of the suture-tissue interaction. The detailed assessment of these aspects will facilitate a deeper understanding of stress distributions, deformation patterns, and failure mechanisms within the suture-tissue system.

## 4. Conclusion

The mechanical behavior and fracture characteristics of sutures used in ophthalmic surgeries (6–0/7–0/8–0 Vicryl, 8–0/9–0/10–0 Nylon, and 9–0/10–0 Polypropylene) were investigated in three different suture configurations (straight, knotted, and looped) under tensile testing. The Vicryl and Nylon sutures exhibited an *S*-shaped load vs. extension response, while the Polypropylene sutures exhibited an *R*-shaped response. The sutures tested in SC demonstrated smooth load vs. extension curves. Whereas, in the KC and LC, a slight load drop was observed in the inelastic region upon full development of the knot. The mechanical properties, *i.e.,* initial stiffness, yield strength, break strength, resilience, and work of rupture, were reduced with the decrease in the gauge diameter of the sutures. The suture’s elongation is strongly influenced by knot administration, along with test configuration, material, structure, and gauge diameter. The Vicryl sutures tested in SC mainly exhibited a high-strain-rate tensile fracture, while Nylon and Polypropylene sutures showed low-strain-rate tensile failure. In contrast, sutures in the KC and LC primarily exhibited tensile-shear failure, resulting from the combined tensile and shear loading due to the presence of the knot.

**Fig 9:**
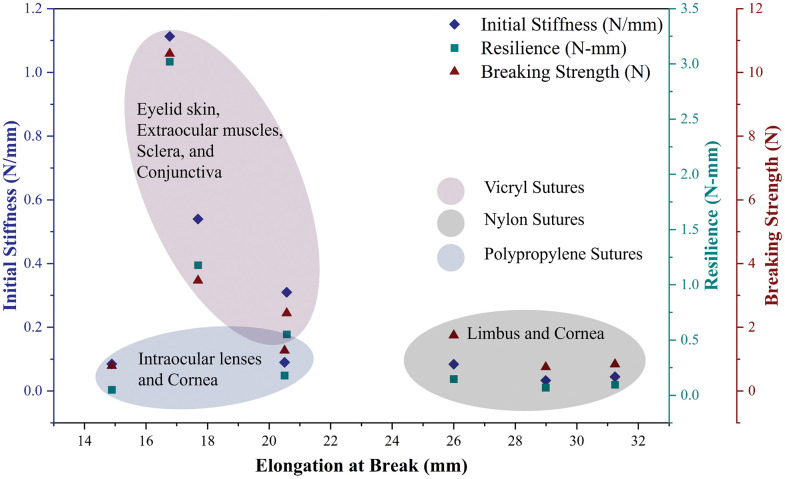
Mechanical properties map for ophthalmic applications.

This study provides a scientific rationale for the appropriate selection of suture materials for various ophthalmic surgeries, based on their mechanical properties, which may enhance ophthalmic surgical outcomes. Fig 9 illustrates the distinct mechanical profiles of suture materials, with the shaded regions indicating their suitability for specific ophthalmic tissues. The evaluated mechanical properties of sutures can be used in (1) ophthalmic surgical interventions to provide a realistic simulation of suture-tissue interactions and (2) enhanced suture design in the future.

## Supporting information

S1 File 7−0 Vicryl Suture in Looped Configuration. Video showing deformation of 7−0 Vicryl suture under looped configuration until fracture.
(MP4)

S2 File 10−0 Nylon Suture in Knotted Configuration. Video showing deformation of 10−0 Nylon suture under knotted configuration until fracture.
(MP4)

S3 File 10−0 Polypropylene Suture in Knotted Configuration. Video showing deformation of 10−0 Polypropylene suture under knotted configuration until fracture.
(MP4)

S4 Data Raw experimental data. Raw data for generating the load vs. Extension curves presented in manuscript and statistical calculations.
(XLSX)

## References

[1] Veeraraghavan R. Wound closure and care in oral and maxillofacial surgery. Oral and maxillofacial surgery for the clinician. Singapore: Springer Nature Singapore. 2021:217–37.

[2] Melles GR, Binder PS. A comparison of wound healing in sutured and unsutured corneal wounds. Arch Ophthalmol. 1990;108(10):1460–9. doi: 10.1001/archopht.1990.01070120108039 2222279

[3] Melles GR, Binder PS, Beekhuis WH, Wijdh RH, Moore MN, Anderson JA, et al. Scar tissue orientation in unsutured and sutured corneal wound healing. Br J Ophthalmol. 1995;79(8):760–5. doi: 10.1136/bjo.79.8.760 7547789 PMC505244

[4] Rubin JA, Runge JJ, Mison M, Mehler S, Echols MS, Lam NKY. Surgery. In: Current Therapy in Avian Medicine and Surgery. Elsevier. 2016:631–68.

[5] Milenković S, Jaković N, Stanković B. Selection of suture material for scleral lacerations. Eur J Ophthalmol. 1996;6(3):327–30. doi: 10.1177/112067219600600318 8908442

[6] Yang Y, Chihaia M, Schulz CB, Kenchington A, Parkin B, MacLean H. 8-0 polyglactin 910 suture in entropion repair: long term follow up and rates of recurrence. Eye. 2023;37(4). 10.1038/s41433-022-01997-5 PMC9998438 35249106

[7] MacDonald JD. Learning to perform microvascular anastomosis. Skull Base. 2005;15(3):229–40. doi: 10.1055/s-2005-872598 16175232 PMC1214708

[8] Lee JH, Chang JH. Suture to limbus distances in eyes with a posterior chamber intraocular lens implanted by scleral fixation. J Cataract Refract Surg. 1993;19(2):278–83. doi: 10.1016/s0886-3350(13)80956-x 8487174

[9] Collin TW, Blyth K, Hodgkinson PD. Cleft lip repair without suture removal. J Plast Reconstr Aesthet Surg. 2009;62(9):1161–5. doi: 10.1016/j.bjps.2008.03.028 19028155

[10] Doi K. Nerves. Flaps and Reconstructive Surgery. 2009:71–9.

[11] Afewerki S, Harb SV, Stocco TD, Ruiz-Esparza GU, Lobo AO. Polymers for surgical sutures. Advanced technologies and polymer materials for surgical sutures. Elsevier. 2023:95–128.

[12] Azhahia Manavalan R, Mukhopadhyay A. Surgical Sutures: Performance, Development and Use. JBBTE. 2008;1:1–36. doi: 10.4028/www.scientific.net/jbbte.1.1

[13] Benicewicz BC, Hopper PK. Review: Polymers for absorbable surgical sutures—part I. J Bioact Compat Polym. 1990;5(4):453–72.

[14] Byrne M, Aly A. The surgical suture. Aesthet Surg J. 2019;39:S67–72. 10.1093/asj/sjz036 30869751

[15] Chu CC. Types and properties of surgical sutures. Biotextiles as Medical Implants. Elsevier Inc. 2013:231–73.

[16] Yag-Howard C. Sutures, needles, and tissue adhesives: a review for dermatologic surgery. Dermatol Surg. 2014;40(Suppl 9):S3–15. 10.1097/01.DSS.0000452738.23278.2d 25158874

[17] Moy RL, Waldman B, Hein DW. A review of sutures and suturing techniques. J Dermatol Surg Oncol. 1992;18(9):785–95. doi: 10.1111/j.1524-4725.1992.tb03036.x 1512311

[18] Kim J-S, Shin S-I, Herr Y, Park J-B, Kwon Y-H, Chung J-H. Tissue reactions to suture materials in the oral mucosa of beagle dogs. J Periodontal Implant Sci. 2011;41(4):185–91. doi: 10.5051/jpis.2011.41.4.185 21954423 PMC3175498

[19] Langley-Hobbs SJ. Sutures and general surgical implants. Feline Soft Tissue and General Surgery. Elsevier Ltd. 2013:105–16.

[20] Tsugawa AJ, Verstraete FJM. Suture materials and biomaterials. Oral and Maxillofacial Surgery in Dogs and Cats. Elsevier Ltd. 2012:69–78.

[21] Afewerki S, Harb SV, Stocco TD, Ruiz-Esparza GU, Lobo AO. Polymers for surgical sutures. Advanced technologies and polymer materials for surgical sutures. Elsevier. 2023:95–128.

[22] He W, Benson R. Polymeric biomaterials. Applied plastics engineering handbook: processing, materials, and applications. Second ed. Elsevier Inc. 2017:145–64.

[23] Javed F, Al-Askar M, Almas K, Romanos GE, Al-Hezaimi K. Tissue reactions to various suture materials used in oral surgical interventions. ISRN Dent. 2012;2012:1–6. 10.5402/2012/762095 PMC3356909 22645688

[24] Kettle C, Dowswell T, Ismail KM. Absorbable suture materials for primary repair of episiotomy and second degree tears. Cochrane Database Syst Rev. 2010;2010(6):CD000006. doi: 10.1002/14651858.CD000006.pub2 20556745 PMC7263442

[25] Narasimhan AK, Rahul TS, Krishnan S. Revisiting the properties of suture materials: an overview. Advanced technologies and polymer materials for surgical sutures. Elsevier. 2023:199–235.

[26] Gillanders SL, Anderson S, Mellon L, Heskin L. A systematic review and meta-analysis: Do absorbable or non-absorbable suture materials differ in cosmetic outcomes in patients requiring primary closure of facial wounds? J Plast Reconstr Aesthet Surg. 2018;71(12):1682–92. doi: 10.1016/j.bjps.2018.08.027 30268743

[27] Dragovic M, Pejovic M, Stepic J, Colic S, Dozic B, Dragovic S, et al. Comparison of four different suture materials in respect to oral wound healing, microbial colonization, tissue reaction and clinical features—randomized clinical study. Clin Oral Investig. 2020;24(4):1527–41. 10.1007/s00784-019-03034-4 31342245

[28] Selvi F, Çakarer S, Can T, Kırlı Topçu Sİ, Palancıoğlu A, Keskin B. Effects of different suture materials on tissue healing. J Istanb Univ Fac Dent. 2016;50(1):35–42. 28955553 10.17096/jiufd.79438 PMC5573451

[29] Kudur MH, Pai SB, Sripathi H, Prabhu S. Sutures and suturing techniques in skin closure. Indian J Dermatol Venereol Leprol. 2009;75(4):425–34. doi: 10.4103/0378-6323.53155 19584482

[30] Thacker JG, Rodeheaver G, Kurtz L, Edgerton MT, Edich RF. Mechanical performance of sutures in surgery. Am J Surg. 1977;133(6):713–5. doi: 10.1016/0002-9610(77)90161-1 326074

[31] Naleway SE, Lear W, Kruzic JJ, Maughan CB. Mechanical properties of suture materials in general and cutaneous surgery. J Biomed Mater Res B Appl Biomater. 2015;103(4):735–42. doi: 10.1002/jbm.b.33171 25045025

[32] Holmlund DE. Physical properties of surgical suture materials: Stress-strain relationship, stress-relaxation and irreversible elongation. Ann Surg. 1976;184(2):189–93. doi: 10.1097/00000658-197608000-00010 952566 PMC1344430

[33] Szabelski J, Karpiński R. Short-term hydrolytic degradation of mechanical properties of absorbable surgical sutures: a comparative study. J Funct Biomater. 2024;15(9). 10.3390/jfb15090273 PMC11432777 39330248

[34] Pacer E, Griffin DW, Anderson AB, Tintle SM, Potter BK. Suture and Needle Characteristics in Orthopaedic Surgery. JBJS Rev. 2020;8(7):e19.00133. doi: 10.2106/JBJS.RVW.19.00133 32649161

[35] Callahan TL, Lear W, Kruzic JJ, Maughan CB. Mechanical properties of commercially available nylon sutures in the United States. J Biomed Mater Res B Appl Biomater. 2017;105(4):815–9. doi: 10.1002/jbm.b.33600 26777335

[36] Chu CC. Mechanical properties of suture materials: an important characterization. Ann Surg. 1981;193(3):365–71. doi: 10.1097/00000658-198103000-00021 6260044 PMC1345078

[37] Alves de Oliveira M, Arcanjo A, Castro F, Fernandes JCH, Fernandes GVO. Evaluating and comparing the tensile strength and clinical behavior of monofilament polyamide and multifilament silk sutures: a systematic review. Surgeries. 2024;5(2):350–66.

[38] Abiri A, Paydar O, Tao A, LaRocca M, Liu K, Genovese B, et al. Tensile strength and failure load of sutures for robotic surgery. Surg Endosc. 2017;31(8):3258–70. doi: 10.1007/s00464-016-5356-1 27928670 PMC5462887

[39] Calfee RP, Boone S, Stepan JG, Osei DA, Thomopoulos S, Boyer MI. Looped versus single-stranded flexor tendon repairs: a cadaveric mechanical study. J Hand Surg Am. 2015;40(5):958–62.e1. doi: 10.1016/j.jhsa.2015.01.035 25801581 PMC4428313

[40] Greenwald D, Shumway S, Albear P, Gottlieb L. Mechanical comparison of 10 suture materials before and after in vivo incubation. J Surg Res. 1994;56(4):372–7. doi: 10.1006/jsre.1994.1058 8152233

[41] Momose T, Amadio PC, Zhao C, Zobitz ME, An KN. The effect of knot location, suture material, and suture size on the gliding resistance of flexor tendons. J Biomed Mater Res (Appl Biomater). 2000;53. 10.1002/1097-4636(2000)53:6<806::aid-jbm23>3.0.co;2-p 11074440

[42] Wang M, Xiang X, Wang Y, Ren Y, Yang L, Zhang Y. Comparison of Tensile Properties and Knot Security of Surgical Sutures: An In Vitro Mechanical Study. Journal of Oral and Maxillofacial Surgery. 2022;80(7):1215–22. 35452601 10.1016/j.joms.2022.03.014

[43] Faulkner BC, Tribble CG, Thacker JG, Rodeheaver GT, Edlich RF. Knot performance of polypropylene sutures. J Biomed Mater Res. 1996;33(3):187–92. doi: 10.1002/(SICI)1097-4636(199623)33:3<187::AID-JBM8>3.0.CO;2-M 8864890

[44] Brauner SC, Berry JL, Pye J, Lee EG, Rhee DJ. Effect of saline conditions on the tensile strength of ophthalmic sutures. Ophthalmic Surg Lasers Imaging. 2011;42(2):148–51. doi: 10.3928/15428877-20101124-01 21117584

[45] Bainbridge JW, Teimory M, Kirwan JF, Rostron CK. A prospective controlled study of a 10/0 absorbable polyglactin suture for corneal incision phacoemulsification. Eye (Lond). 1998;12 (Pt 3a):399–402. doi: 10.1038/eye.1998.94 9775239

[46] Shuttleworth GN, Vaughn LF, Hoh HB. Material properties of ophthalmic sutures after sterilization and disinfection. J Cataract Refract Surg. 1999;25(9):1270–4. doi: 10.1016/s0886-3350(99)00156-x 10476513

[47] Rennie L, Fleming W, Clark D, Ellerton C, Bosanquet R. Some mechanical properties of 10/0 monofilament nylon sutures. Eye (Lond). 1994;8 (Pt 3):343–5. doi: 10.1038/eye.1994.71 7958044

[48] Brooks SE. Securing extraocular muscles in strabismus surgery: laboratory analysis of biomechanical parameters related to the suture. Journal of AAPOS. 2017;21(6):457–9.e1. 28989101 10.1016/j.jaapos.2017.03.015

[49] Jones R, Dean W. Basic microsurgical skills: suturing. Community Eye Health. 2023;36(120):8–10. PMC10762710 38178817

[50] Kaur Randhawa R, Dubey T, Pansuriya I, Mishra T, Tanwar M, Kumar A, et al. Assessment of the Mechanical Properties of Different Suture Materials for Oral Surgery: An In Vitro Tensile Strength Study. Cureus. 2024;16(8):e65952. doi: 10.7759/cureus.65952 39221394 PMC11365713

[51] Hennessey DB, Carey E, Simms CK, Hanly A, Winter DC. Torsion of monofilament and polyfilament sutures under tension decreases suture strength and increases risk of suture fracture. J Mech Behav Biomed Mater. 2012;12:168–73. 22762905 10.1016/j.jmbbm.2012.02.001

[52] Taysi AE, Ercal P, Sismanoglu S. Comparison between tensile characteristics of various suture materials with two suture techniques: an in vitro study. Clin Oral Investig. 2021;25(11). 10.1007/s00784-021-03943-3 33851242

[53] Polykandriotis E, Daenicke J, Bolat A, Grüner J, Schubert DW, Horch RE. Individualized Wound Closure-Mechanical Properties of Suture Materials. J Pers Med. 2022;12(7):1041. doi: 10.3390/jpm12071041 35887538 PMC9316899

[54] Silver E, Wu R, Grady J, Song L. Knot security- how is it affected by suture technique, material, size, and number of throws? Journal of Oral and Maxillofacial Surgery. W.B. Saunders. 2016:1304–12. 10.1016/j.joms.2016.02.004 26979258

[55] Aktı A, Kaya DI. Evaluation of tensile strength of surgically absorbable suture materials used in oral surgery after immersion in different beverages: an in vitro study. Materials. 2024;17(14). 10.3390/ma17143586 PMC11278735 39063878

[56] Anushya P, Ganesh SB, Jayalakshmi S. Evaluation of tensile strength of surgical absorbable and nonabsorbable suture materials after immersion in different fruit juices: An in vitro study. J Adv Pharm Technol Res. 2022;13(Suppl 1):S108–11. doi: 10.4103/japtr.japtr_267_22 36643124 PMC9836114

[57] Abullais SS, Alosman SS, Alqahtani SM, Khan AA, Nahid R, Basheer SA. Effect of common mouthwashes on mechanical properties of suture materials used in dental surgeries: A laboratory experiment. Polymers (Basel). 2022;14(12). 10.3390/polym14122439 PMC9227470 35746015

[58] Alsarhan M, Alnofaie H, Ateeq R, Almahdy A. The Effect of Chlorhexidine and Listerine® Mouthwashes on the Tensile Strength of Selected Absorbable Sutures: An In Vitro Study. Biomed Res Int. 2018;2018:8531706. doi: 10.1155/2018/8531706 30539024 PMC6257908

[59] Bennett RG. Selection of wound closure materials. J Am Acad Dermatol. 1988;18(4 Pt 1):619–37. doi: 10.1016/s0190-9622(88)70083-3 3286691

[60] Chittajallu SNSH, Gururani H, Jakati S, Basu S, Vaddavalli PK, Tse KM, et al. Investigation of mechanical strength and structure of corneal graft-host junction. Heliyon. 2024;10(10):e30871. doi: 10.1016/j.heliyon.2024.e30871 38784531 PMC11112333

[61] Zhao C, Hsu C-C, Moriya T, Thoreson AR, Cha SS, Moran SL, et al. Beyond the square knot: a novel knotting technique for surgical use. J Bone Joint Surg Am. 2013;95(11):1020–7. doi: 10.2106/JBJS.K.01525 23780540 PMC3748986

[62] Rawal A, Kumar R, Saraswat H. Tensile mechanics of braided sutures. Textile Research Journal. 2012;82(16):1703–10. doi: 10.1177/0040517512445340

[63] Ben Abdessalem S, Debbabi F, Jedda H, Elmarzougui S, Mokhtar S. Tensile and Knot Performance of Polyester Braided Sutures. Textile Research Journal. 2009;79(3):247–52. doi: 10.1177/0040517508094090

[64] Martin DA, Boron K, Obstalecki M, Kurath P, Horn GP. Feasibility of Knots to Reduce the Maximum Dynamic Arresting Load in Rope Systems. J dynamic behavior mater. 2015;1(2):214–24. doi: 10.1007/s40870-015-0015-5

[65] Zha S, Lan H, Huang H. Review on lifetime predictions of polyethylene pipes: Limitations and trends. International Journal of Pressure Vessels and Piping. 2022;198:104663. doi: 10.1016/j.ijpvp.2022.104663

[66] Tan CJ, Andriyana A, Ang BC, Wong D. Mechanical deformation and fracture mechanisms of polymeric fibres from the perspective of fractography – A review. European Polymer Journal. 2020;137.

[67] Sedláček D, Stöhr A. Cut resistance of climbing ropes – a comparative analysis of existing measurement methods and a simulated accident. Eng Fail Anal. 2024;157.

[68] Hearle JWS, Lomas B, Cooke WD. Ductile tensile fracture: Nylon, polyester, polypropylene, etc. Atlas of fibre fracture and damage to textiles. 2 ed. Elsevier. 1998:42–9.

[69] Saville BP. Strength and elongation tests. Physical Testing of Textiles. Elsevier. 1999:115–67. doi: 10.1533/9781845690151.115

[70] Chen LE, Seaber AV, Urbaniak JR. Comparison of 10-0 Polypropylene and 10-0 Nylon Sutures in Rat Arterial Anastomosis. Microsurgery. 328–33. 10.1002/micr.1920140508 8332053

[71] Kim J-C, Lee Y-K, Lim B-S, Rhee S-H, Yang H-C. Comparison of tensile and knot security properties of surgical sutures. J Mater Sci Mater Med. 2007;18(12):2363–9. doi: 10.1007/s10856-007-3114-6 17569012

[72] Semjonow A, Brandt M, Reul H, Rathert P. Suture surface and suture strength of polypropylene monofilaments. Biomed Tech (Berl). 1993;38(1–2):21–4. doi: 10.1515/bmte.1993.38.1-2.21 8461445

[73] van Rijssel EJ, Trimbos JB, Booster MH. Mechanical performance of square knots and sliding knots in surgery: comparative study. Am J Obstet Gynecol. 1990;162(1):93–7. doi: 10.1016/0002-9378(90)90828-u 2154104

[74] Briceno-Lopez C, Burguera-Giménez N, García-Domene MC, Díez-Ajenjo MA, Peris-Martínez C, Luque MJ. Corneal Edema after Cataract Surgery. Journal of Clinical Medicine. 2023;12. 10.3390/jcm12216751 PMC10647590 37959216

[75] Sharma N, Singhal D, Nair SP, Sahay P, Sreeshankar SS, Maharana PK. Corneal edema after phacoemulsification. Indian J Ophthalmol. 2017;65(12):1381–9. doi: 10.4103/ijo.IJO_871_17 29208818 PMC5742966

[76] Huang Y, Cadet ER, King MW, Cole JH. Comparison of the mechanical properties and anchoring performance of polyvinylidene fluoride and polypropylene barbed sutures for tendon repair. J Biomed Mater Res B Appl Biomater. 2022;110(10):2258–65. doi: 10.1002/jbm.b.35074 35674273 PMC9546200

[77] Karaca E, Hockenberger AS. Analysis of the fracture morphology of polyamide, polyester, polypropylene, and silk sutures before and after implantation in vivo. J Biomed Mater Res B Appl Biomater. 2008;87B(2):580–9. 18506829 10.1002/jbm.b.31136

[78] Alsarhan MA. A systematic review of the tensile strength of surgical sutures. J Biomater Tissue Eng. 2020;9(11):1467–76.

